# Intra‐Annual and Interannual Dynamics of Evaporation Over Western Lake Erie

**DOI:** 10.1029/2020EA001091

**Published:** 2020-11-23

**Authors:** Changliang Shao, Jiquan Chen, Housen Chu, Carol A. Stepien, Zutao Ouyang

**Affiliations:** ^1^ Institute of Agricultural Resources and Regional Planning Chinese Academy of Agricultural Sciences Beijing China; ^2^ Center for Global Change and Earth Observations Michigan State University East Lansing MI USA; ^3^ Climate and Ecosystem Sciences Division Lawrence Berkeley National Laboratory Berkeley CA USA; ^4^ NOAA Pacific Marine Environmental Laboratory Seattle WA USA

**Keywords:** evaporation, extreme climate, Great Lakes, ice cover, latent heat flux, Penman model

## Abstract

Evaporation (*E*) is a critical component of the water and energy budget in lake systems yet is challenging to quantify directly and continuously. We examined the magnitude and changes of *E* and its drivers over Lake Erie—the shallowest and most southern lake of the Laurentian Great Lakes. We deployed two eddy‐covariance tower sites in the western Lake Erie Basin—one located nearshore (CB) and one offshore (LI)—from September 2011 through May 2016. Monthly *E* varied from 5 to 120 mm, with maximum *E* occurring in August–October. The annual *E* was 635 ± 42 (±SD) mm at CB and 604 ± 32 mm at LI. Mean winter (October–March) *E* was 189 ± 61 mm at CB and 178 ± 25 mm at LI, accounting for 29.8% and 29.4% of annual *E*. Mean daily *E* was 1.8 mm during the coldest month (January) and 7.4 mm in the warmest month (July). Monthly *E* exhibited a strong positive linear relationship to the product of wind speed and vapor pressure deficit. Pronounced seasonal patterns in surface energy fluxes were observed with a 2‐month lag in *E* from *R*_n_, due to the lake's heat storage. This lag was shorter than reports regarding other Great Lakes. Difference in *E* between the offshore and nearshore sites reflected within‐lake spatial heterogeneity, likely attributable to climatic and bathymetric differences between them. These findings suggest that predictive models need to consider lake‐specific heat storage and spatial heterogeneity in order to accurately simulate lake *E* and its seasonal dynamics.

## Key Points

- The maximum lake evaporation occurred in August–October, with one third in winter over the annual evaporation
- Monthly evaporation showed a positive linear relationship with vapor pressure deficit and temperature but not correlated with wind speed
- Pronounced seasonal patterns in the surface energy fluxes were observed with a 2‐month lag in evaporation from net radiation

## 1 Introduction

Evaporation (*E*) is an essential component of the water and energy balance over lakes (Gronewold & Stow, 2014; Oki & Kanae, 2006). Increased lake evaporation has been responsible for record decreases in water levels in the Laurentian Great Lakes and across North America (Gronewold et al., 2016; Sellinger et al., 2008; Spence et al., 2013), along with the disappearance of inland lakes in Mongolia (Tao et al., 2015). Global warming causes higher air and lake water temperatures (Austin & Colman, 2007), as well as reductions in ice cover (both extent and duration) (Duguay et al., 2006; Wang et al., 2012), leading to elevated *E*. Thermodynamically, *E* acts as negative feedback to dampen further warming. Large lakes can serve to modulate local weather and climate through mesoscale circulations, such as via alterations in surface heat fluxes, moisture, and lake effects on storms and lake breezes (Long et al., 2007). A better understanding of *E* processes and their underlying mechanisms is critical for improving model estimates of lake *E* to more accurately predict feedbacks with climate change (Blanken et al., 2011; Fujisaki‐Manome et al., 2017; Gronewold et al., 2016; Spence et al., 2013).

Direct measurements of lake *E*, especially over large lakes, have been sparse due to technical challenges and accessibility (Blanken et al., 2011). Often, lake *E* is estimated based on evaporation pans, inferred from water or energy balances (McJannet et al., 2012), or calculated by combining energy balance and mass transfer methods (Derecki, 1976; Schertzer, 1987). Conventional methods have relied on sparse water depth observations and often on shore‐based weather stations (Jensen, 2010). Improvements in the eddy covariance (EC) technique in recent decades has enabled direct and continuous measurements of *E* over lakes (Blanken et al., 2000; Spence et al., 2013). Direct measurements of *E* have proven to be critical for evaluating and improving the models (Charusombat et al., 2018; Fujisaki‐Manome et al., 2017).

Year‐round and long‐term lake *E* measurements are fundamental for understanding these processes and to constrain the models of lake hydrologic cycles. However, such data sets rarely are available due to accession and logistic challenges posed by lake environments (Charusombat et al., 2018; McMahon et al., 2013). Most studies have relied on short‐term campaigns restricted to the warm season, which may bias estimates of seasonal variation and annual sums of lake *E*. In deep lakes, turnover of the water column during the fall plays an important role in regulating the seasonal dynamics of *E* (Jensen, 2010). On the other hand, shallow lakes have been found to exhibit conservative evaporative water loss during the cool (winter) season due to lack of convectional water overturn (Granger & Hedstrom, 2011; Spence et al., 2003). Estimates of cool season *E* in shallow lakes thus mostly have relied on models and climatological observations made at shore‐based weather stations (McMahon et al., 2013; Morton, 1983). However, most hydroclimate models require or perform more robustly using forcing data that are directly measured over lakes (e.g., Fujisaki‐Manome et al., 2017; Huang et al., 2011). Fujisaki‐Manome et al. (2017) stressed the criticalness of directly measuring *E* in relation to meteorological variables over lakes year‐round, as accomplished in the present study.

Substantial time lags between lake *E* and net radiation (*R*_n_) have been reported, attributed to the water column's heat storage capacity. Deep lakes, with their larger heat capacity, experience larger seasonal lags in evaporation than small lakes. For example, Lofgren and Zhu (2000) found a five to 6‐month delay between peak energy input and lake *E* over most of the Great Lakes based on estimates from remotely sensed lake surface temperatures. Yet Lake Erie and shallow parts of other lakes had shorter lags and did not maintain high *E* in the late fall and winter seasons. Liu et al. (2012) revealed that *E* exceeded *R*_n_ during the cool season, with the energy deficit supplied by the release of heat that had been stored during the warm season from a reservoir in Mississippi. Rouse et al. (2003) and Blanken et al. (2003) reported that 70–90% of total *E* occurred after August in the northern Great Slave Lake in Canada, with only a small amount of *E* in the summer. Therefore, the lake was an energy sink during the summer and an energy source in the fall/winter, inducing a large seasonal thermal lag to the regional climate (Long et al., 2007). Here, this thermal‐lag concept is key to understanding and accurately modeling the temporal dynamics of lake *E*.

Evaporation from the Great Lakes, especially over shallow Lake Erie (mean depth = 19 m, maximum depth = 64 m [in the eastern basin]), has been identified as one of the least understood and poorly constrained processes in the Great Lakes hydrologic cycle. Among the Great Lakes, Lake Erie possessed the highest relative uncertainty (41–101%) in the water budget, outlining the knowledge deficit prior to our study. Lake *E*, which had not been directly measured but instead modeled (Neff & Nicholas, 2005), was one of the largest sources of uncertainties (i.e., up to 35% for monthly *E*). Recent studies have highlighted the importance of direct and continuous measurements of lake *E* in improving models to accurately estimate water and energy fluxes for the Great Lakes (Charusombat et al., 2018; Fujisaki‐Manome et al., 2017). Although studies existed for some of the Great Lakes, long‐term measurements of *E* and the length of the lag between peak *R*_n_ and *E* for Lake Erie were not known or reported previously, to the best of our knowledge.

We studied the process and regulating factors of *E* over Lake Erie using the EC technique at two over‐lake sites—one offshore and one nearshore—from September 2011 to May 2016. We hypothesized that greater *E* occurred after the warm season due to heat storage in Lake Erie. Our specific study objectives were to determine (1) the intra‐annual variation of *E*, including across the cool and warm seasonal periods and their contributions to the annual sums; (2) the climatic variables regulating the interannual variations of *E*; and (3) the differences of *E* and the climatic forcing between the two over‐lake sites.

## 2 Sites and Methods

### 2.1 Sites Description

Lake Erie is one of the Laurentian Great Lakes and is the fifth largest lake in North America (174 m A.S.L.), with a surface area of 25.7 × 10^3^ km^2^, a volume of 545 km^3^, and a mean depth of 19 m. Lake Erie is the shallowest, most southern, and warmest in summer; has the most ice coverage in winter; and is the most biologically productive Laurentian Great Lake (Bolsenga & Herdendorf, 1993). The western basin (where the current study was focused) has a relatively shallow mean water depth of 7.4 m and is highly eutrophic, nutrient rich, and turbid, with the greatest algae productivity (Bolsenga & Herdendorf, 1993; Ouyang et al., 2017). Lake Erie's primary inflow is the Detroit River, and its main outflow is the Niagara River. It typically has the warmest water surface temperature in summer among the Great Lakes and is the first to freeze in the winter. The western basin is naturally separated from the deeper central basin to the east by bedrock islands, reefs, and shoals. Western Lake Erie is characterized by strong winds with northwest to east‐west directionality. It is subject to lake‐effect snow beginning in November, when the cold winds of winter pass over its warm waters (Fujisaki‐Manome et al., 2017). The snow generally melts after February, although there are exceptions due to climate variation. Based on these descriptions, we here defined the warm season as April–September and the cool season as October–March.

**Figure 1:**
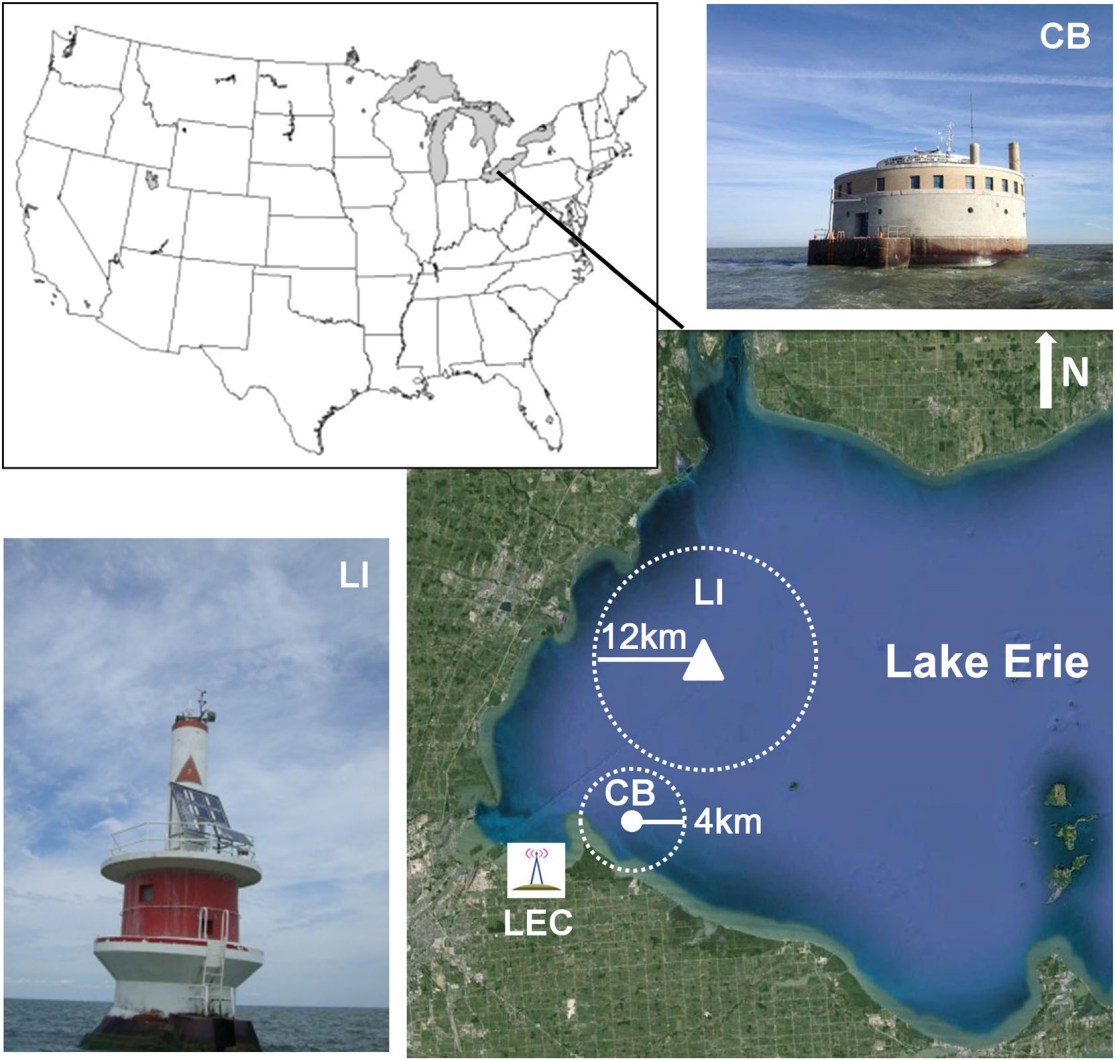
Locations of the eddy covariance systems at the CB (N41.7167, W83.2667) and LI sites (N41.8314, W83.2006) in western Lake Erie. Real time data were transmitted via spread spectrum radio (RF450, Campbell Scientific Inc.) back to our data server at the University of Toledo's Lake Erie Center (LEC; the greatest distance was ~25 km from the tower).

Two permanent EC flux stations, each equipped with wireless transmission instruments, were installed in western Lake Erie for this study, one at the crib site (CB) (i.e., the water intake of the City of Toledo Division of Water Treatment) and the other at the light site (LI) on the NOAA No. 2 light buoy (https://www.glerl.noaa.gov/metdata/tol2/) (Figure 1). The CB and LI sites are located ~4 km (41°41′57″N, 83°15′34″W) and ~12 km (41°49′32″N, 83°11′37″W) from the nearest shore, with mean water depths of ~5.1 and 7.5 m, respectively.

### 2.2 Eddy‐Covariance and Meteorological Measurements

Open‐path EC systems with affiliated meteorological sensors were installed from September 2011 to June 2016 at about 15 m above the water surface to measure the net exchange of latent heat (*LE*), sensible heat (*H*), and CO_2_ fluxes between the lake and the atmosphere at the CB and LI sites. Each EC system included an infrared gas analyzer (IRGA, Model LI‐7500A, LI‐COR, Lincoln, NE, USA) and a CSAT3 three‐dimensional sonic anemometer (Campbell Scientific Inc. [CSI], Logan, UT, USA). Three‐dimensional wind velocities, sonic temperatures, and H_2_O and CO_2_ concentrations were sampled at 10 Hz frequency, and the raw time series (*TS*) data were stored in CR3000 dataloggers (CSI). IRGA was calibrated before the start of the campaign in 2011 and was rotated with a newly calibrated IRGA unit during annual maintenance trips. Micrometeorological variables included photosynthetically active radiation (*PAR*) (LI‐190, LI‐COR), net radiation (*R*_n_) (CNR4, Kipp and Zonen, Delft, Netherlands), relative humidity (*RH*), and air temperature (*T*_a_) (HMP45C, CSI), which were measured at the same height as the EC system. Water surface and column temperatures were measured using an IRR‐P Infrared thermometer (CSI) and a profile of CS107 probes (CSI) at 0.2‐, 0.5‐, 1.0‐, 2.0‐, and 4.0‐m depths. Rainfall was measured with a tipping bucket rain gauge (TE‐525, CSI). The 10‐Hz *TS* data and half‐hourly meteorological data were transmitted back to our data server located at the University of Toledo's Lake Erie Center (LEC) in Oregon, Ohio (41°41′18″N, 83°23′58″W), located ~10 and ~25 km from the CB and LI sites (Figure 1), via a spread spectrum radio (RF450 [CSI] with a 14201 Yagi directional antenna). NOAA's real‐time imagery (http://www.glerl.noaa.gov/metdata/tol2/) at the LI site and MODIS imagery (http://www.glerl.noaa.gov/data/pgs/glice) at the CB site were used to determine ice‐present periods at the lake surface, including snow cover.

### 2.3 Flux Calculation, QA/QC, and Gap Filling

The program EdiRe (University of Edinburgh, v1.5.0.32) was used to calculate the half‐hourly *LE* and *H*, following the workflow of Chu et al. (2014, 2015). The planar fit method was applied to rotate the three velocity components into the mean streamline coordinate system, and time lags between measured scalars and vertical velocity were corrected. The u* threshold was set to 0.10 m s^−1^ to filter out periods with low turbulence conditions. The source area of each half‐hourly flux was calculated using an analytical footprint model (Kormann & Meixner, 2001) and used to flag and filter periods when <80% of the measured fluxes originated from the lake (see Shao et al., 2015, for more data QA/QC and processing). Altogether, *H* data that passed the quality checks described above totaled 62% at the CB site and 55% for LI, with 51% and 40% of the *LE* data overall passing (Table 1).

**Table 1:** Cumulative Flux Footprint Contributions From the Areas Within 4,000, 2,000, and 1,000 m Radii Around the Towers, the Available Data Ratio for Latent Heat (*LE*) and Sensible Heat (*H*) Fluxes, and the Energy Balance Ratios (*EBR*) at the CB and LI Sites

Site	Footprint (%)			Good data (%)		EBR
	4,000	2000	1,000	*LE*	*H*	
CB	80.25	68.32	49.72	50.55	62.46	0.90
LI	80.51	73.66	59.98	40.30	55.15	0.92

The energy balance ratio (*EBR*) was used to evaluate closure over an entire year to assess performance of the EC system (Wilson et al., 2002):

(1) $$\mathrm{EBR}=\frac{\sum \left(\mathrm{LE}+H\right)}{\sum \left(R_{n}-G\right)},$$

where *G* is the water heat storage calculated from the temperature profiles (Shao et al., 2008). The EBR was 0.90 and 0.92 at the CB and LI sites, which is higher than the 0.84 ± 0.20 range reported across 173 site‐years of EC data from terrestrial ecosystems (Stoy et al., 2013), suggesting the robustness of EC measurements from the two tower sites.

The marginal distribution sampling (MDS) method (Reichstein et al., 2005) was adopted to fill the data gaps of *LE* and *H*. This was done because (1) fluxes for the lake ecosystems were found to be less coupled with the biological sources/sinks than in terrestrial ecosystems (Vesala et al., 2006) and (2) the MDS approach took advantage of the autocorrelation structure in the flux data and incorporated self‐dependency in filling the gaps (Reichstein et al., 2005), providing a robust approach for gap filling and integrating the time series to provide daily, monthly, or annual sums. Briefly, the gaps of fluxes were filled using the following steps: (1) Linear interpolation was applied to gaps <1.5 hr; (2) remaining gaps were filled with mean half‐hourly values made under similar micrometeorological conditions (difference in *PAR ≤* 100 μmol m^−2^ s^−1^, *VPD* [vapor pressure deficit] *≤* 0.5 kPa, and *T*_a_ *≤* 2.5°C) within a window of 7 days before or after each gap; and (3) if micrometeorological data were unavailable, gaps were filled with the mean diurnal values from a window of 7 days before or after each gap. If no data were available for Steps 2 and 3, the window size was progressively expanded to 14 or 28 days to fill the gap (Shao et al., 2015).

### 2.4 Footprint Climatology and Uncertainty Analyses

To locate the source areas contributing to the measured fluxes over the study period (i.e., footprint climatology), we calculated the cumulative footprint contributions from the areas within 1‐, 2‐, and 4‐km distances from the towers. The footprint climatologies showed that 50%, 68%, and 80% for CB and 60%, 74%, and 81% for LI sites were contributed by the areas within a 1‐, 2‐, and 4‐km radius of the towers, respectively. Because CB and LI towers were located more than 4 and 12 km away from the nearest shore, the influences of land area on the measured fluxes were expected to be marginal at the CB site and negligible at the LI site.

The uncertainties of annual *E* were quantified following Aurela et al. (2002). Briefly, the random error for each half‐hourly flux was estimated following Richardson and Hollinger (2007). The uncertainties originating from the gap‐filling procedures were quantified based on Reichstein et al. (2005). When calculating the annual flux, we propagated the aforementioned half‐hourly uncertainties through Monte Carlo simulations (*N* = 1,000, 95% confidence intervals) into the annual uncertainties. On average, the uncertainty level of the annual *E* was around ±3.7%, corresponding to around ±25 mm year^−1^.

### 2.5 Model Calculation

The Penman approach has been widely used in estimating open‐water *E* due to its simplicity and good performance at a monthly and daily scale (McMahon et al., 2013). We used the model estimate to demonstrate the potential divergence between the measured *E* and a common method used to model it. The combination model of the Penman approach (Equation 16 in Penman, 1948) was adopted to calculate daily *E* using the meteorological data from the LI site as follows:

(2) $$E_{\mathrm{Pen}}=\frac{\Delta }{\Delta +\gamma }\frac{R_{n}}{\lambda }+\frac{\gamma }{\Delta +\gamma }\;E_{a},$$

where 𝐸_Pen_ is the daily open‐water evaporation (mm day^−1^); 𝑅_n_ is the net radiation at the water surface (MJ m^−2^ day^−1^); 𝐸_a_ (mm day^−1^) is a function of wind speed, saturation vapor pressure, and average vapor pressure (kPa); Δ is the slope of the vapor pressure curve (kPa °C^−1^) at air temperature (*T*_a_, °C); γ is the psychrometric constant (kPa °C^−1^); and 𝜆 is the latent heat of vaporization = 2.45 MJ kg^−1^ at 20°C (Allen et al., 1998).

(3) $$\Delta =\frac{4098\left[0.6108\exp\left(\frac{17.27T_{a}}{T_{a}+237.3}\right)\right]}{{\left(T_{a}+237.3\right)}^{2}},$$

(4) $$\gamma =0.00163\;\frac{p}{\lambda },$$

where 𝑝 is atmospheric pressure at elevation 𝑧 m (Equation 7 in Allen et al., 1998)

(5) $$p=101.3{\left(\frac{293-0.0065\;\text{Elev}}{293}\right)}^{5.26}.$$

### 2.6 Data Analysis

Regional long‐term climatology data (e.g., 30‐ and 100‐year mean air temperatures and annual rainfall) were obtained through the National Climatic Data Center of the National Oceanic and Atmospheric Administration, USA. Three weather stations located in Bowling Green (N41.3831, W83.6108, 1893–2015), Fremont (N41.3331, W83.1189, 1901–2015), and Toledo Express Airport (N41.5883, W83.8014, 1955–2015) in Ohio were used because they were located less than 30 km from our sites and had long‐term records (Chu et al., 2014). To determine the diel changes of energy fluxes (Figure 4), all of the quality data were combined into 30‐min averages on a monthly scale. These served to reduce the sampling error associated with individual flux measurements that can result from the intermittent natural states of turbulence caused by horizontal transport across large sunny and shaded patches (Shao et al., 2017). The generalized linear model (GLM) was used to conduct regression analysis. Stepwise regression was used to analyze binary linear regressions of *E* with climatic variables. Gap‐filling procedures and statistical analyses were accomplished using the *R* language (*R* Development Core Team, 2013, Version 3.0.0).

## 3 Results

### 3.1 Lake Microclimate

**Figure 2:**
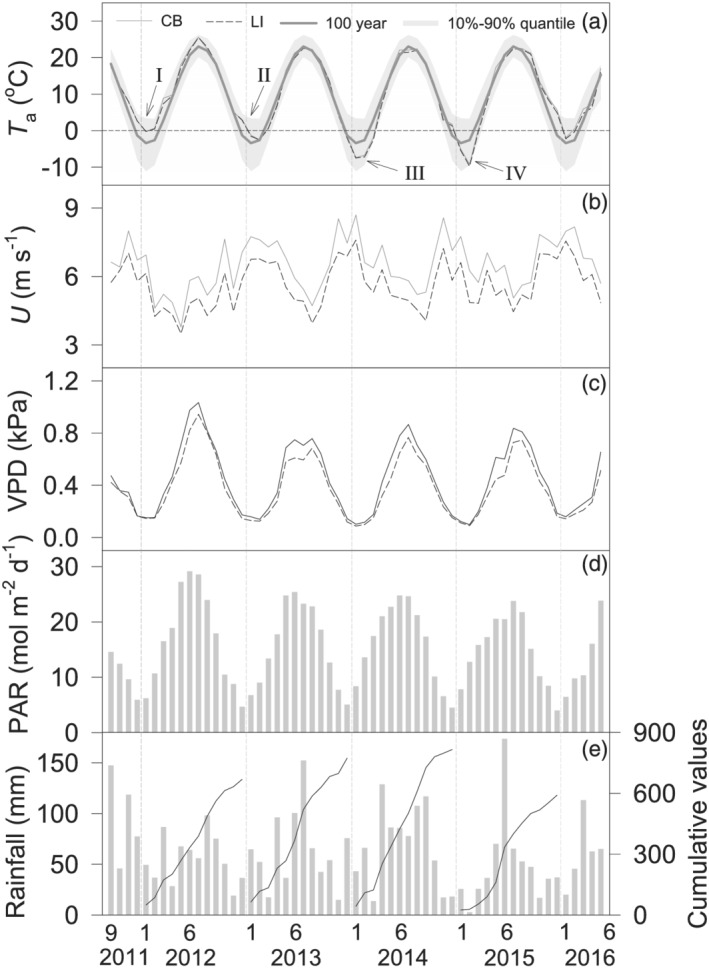
Monthly mean (a) air temperature (*T*_a_), (b) wind speed (*U*), (c) vapor pressure deficit (*VPD*), (d) photosynthetically active radiation (*PAR*), and total and cumulative (e) rainfall measured ~15 m above the water surface at the CB and LI sites over Lake Erie from September 2011 to May 2016. Long‐term (1893–2015) average (gray lines) and its 90% quantile intervals (gray shaded areas) are presented in (a).

Monthly *T*_a_ values above the water surface were similar between the CB and LI sites. Substantial interannual *T*_a_ variation occurred among the winters evaluated here. The five consecutive winters (November–March) had respective mean monthly *T*_a_ values of 4.0°C, 1.0°C, −2.6°C, −1.8°C, and 3.8°C at the CB site and 3.6°C, 0.9°C, −3.0°C, −2.3°C, and 3.1°C at the LI site. Compared to the 122‐year (1894–2015) mean monthly *T*_a_ of 0.1°C, the first, second, and fifth winters were warmer, while the third and fourth winters were cooler (Figure 2a). The *T*_a_ difference between the warmest winter (2011–2012) and the coldest winter (2013–2014) was 6.8°C. Accordingly, the first, second, and fifth years had higher annual *T*_a_ values, while the third and fourth years had lower annual *T*_a_ values than the 122‐year mean of 10.1°C.

The monthly mean wind speed (*U*) during the winter months was 7.1 m s^−1^ at CB and 6.2 m s^−1^ at the LI site but reached 15.0 m s^−1^ during synoptic weather events, providing substantial turbulent mixing (Figure 2b). Both *VPD* and PAR showed seasonal patterns similar to that of *T*_a_. Winter PAR was 9.8, 8.5, 10.4, 9.5, and 7.8 mol m^−2^ day^−1^ at both sites during the five consecutive years (Figures 2c and 2d). Rainfall displayed evident interannual variation, ranging from 660 mm in 2015 to 880 mm in 2011, excluding snowfall during freezing conditions when the rain gauge failed to operate. Monthly rainfall peaked at 148 mm in September 2011, 152 mm in July 2014, and 174 mm in June 2015. Annual rainfall totaled 880, 770, 800, 660, and 740 mm during the five consecutive years, in comparison to the 30‐year mean annual precipitation of 840 mm (including rainfall and snowfall) for the region. The first and third years experienced relatively higher annual rainfall but had contrasting winter *T*_a_.

NOAA's real‐time camera and MODIS imagery recorded longer ice‐present durations (including snow cover) in the third and fourth winters. The first winter experienced a much shorter ice‐present period, with nearly no identified ice cover at the sites (Table 2). The longest ice‐present period occurred in the third winter (2013–2014) and lasted for 117 and 114 days at the CB and LI sites, respectively. The differences between the longest and shortest durations of the ice‐present periods were 114 days for both the CB and LI sites, across the 5 years.

**Table 2:** Dates of Ice‐on, Ice‐off, and Ice‐Present Periods (Days) at the CB and LI Sites in Western Lake Erie

	2011–2012 (first winter)		2012–2013 (second winter)		2013–2014 (third winter)		2014–2015 (fourth winter)		2015–2016 (fifth winter)	
	CB	LI	CB	LI	CB	LI	CB	LI	CB	LI
Ice‐on date	21 Jan (21) 2012	Not frozen	22 Jan (22) 2013	24 Jan (24) 2013	1 Dec (345) 2013	7 Dec (351) 2013	6 Jan (6) 2015	5 Jan (5) 2015	7 Jan (7) 2016	12 Jan (12) 2016
Ice‐out date	23 Jan (23) 2012	Not frozen	13 Mar (72) 2013	27 Mar (86) 2013	27 Mar (86) 2014	31 Mar (90) 2014	15 Mar (74) 2015	27 Mar (86) 2015	15 Feb (46) 2016	20 Feb (51) 2016
Ice‐present period	3	0	51	63	117	114	69	82	40	22^a^

*Note*. For CB, the ice‐present period was obtained from the NOAA website (http://www.glerl.noaa.gov/data/pgs/glice); for LI, the ice‐present period was observed directly via a three‐direction real‐time camera operated by NOAA‐GLERL (http://www.glerl.noaa.gov/res/recon/). Numbers in parentheses indicate the day of the year.

^a^ No ice was present from 27 January to 13 February 2016

### 3.2 Diurnal Variation in Lake *E*

**Figure 3:**
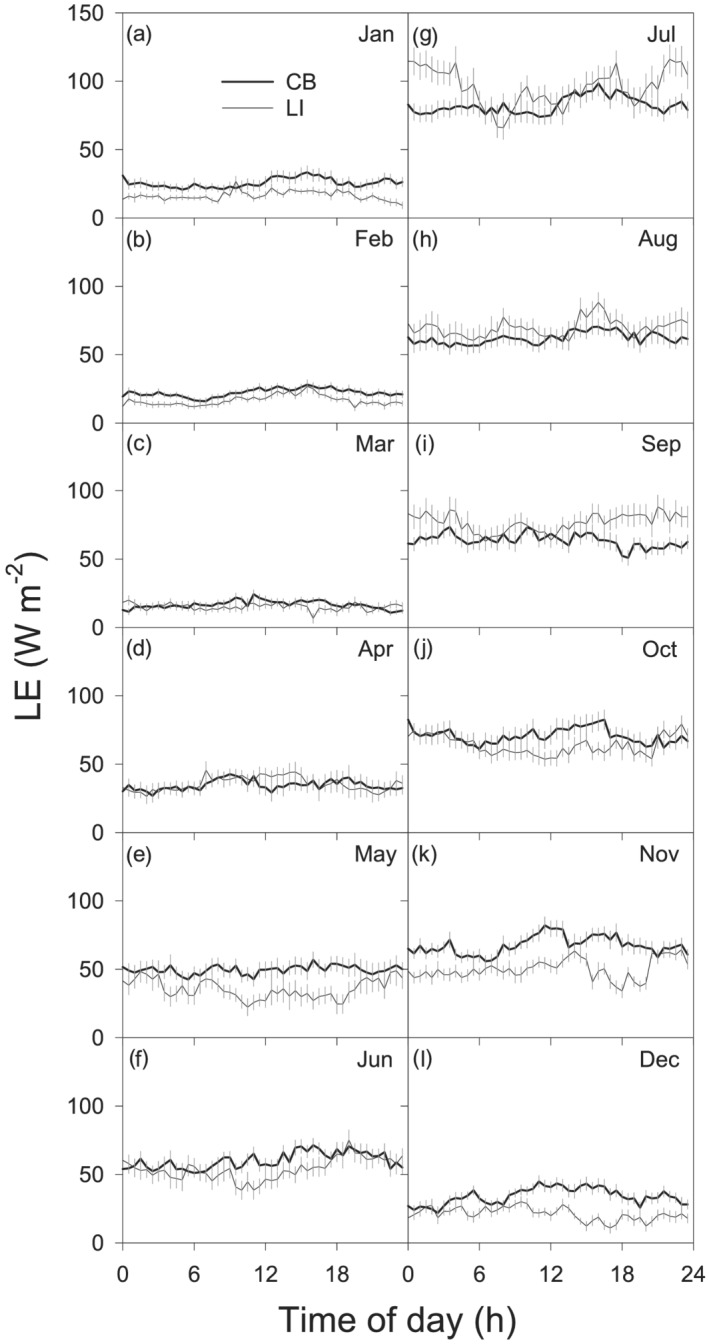
Monthly average diurnal mean latent heat flux (*LE*) at the CB and LI sites in western Lake Erie. Data covered the entire measurement period. Charts a‐l show results from January–December, respectively. Data are presented in local standard time (UTC‐5).

Diel *E* changes revealed marginal variations between day versus night, along with obvious differences among months (Figure 3). Maximum *E* (~115 W m^−2^, equal to around 0.21 mm, 30 min^−1^) was observed from the afternoon through early morning of the following day in July at the LI site, whereas minimum *E* (~10 W m^−2^, equal to around 0.02 mm, 30 min^−1^) occurred from late afternoon through early morning from January to March at the CB site. The daily peak of *E* was greatest from July to September, although it also was notable in October and November. The daily peak of *E* was lowest during January and February (Figures 3a and 3b). Slightly higher *E* was observed at night during May–October at LI, particularly during the months of May–July and October (Figure 3).

### 3.3 Seasonal Variation in Lake *E*

**Figure 4:**
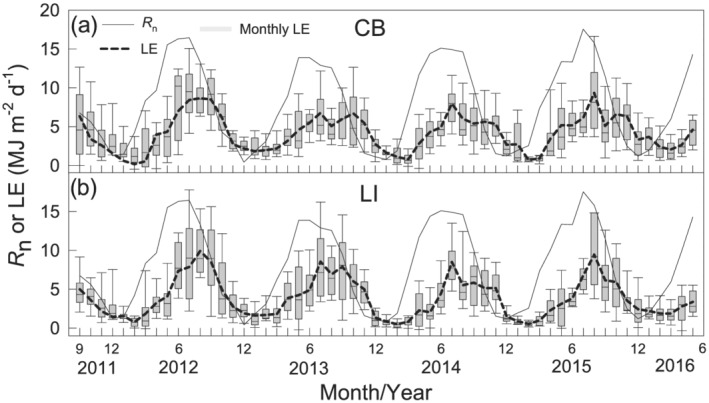
Monthly net radiation (*R*_n_) and latent heat flux (*LE*) at the two Lake Erie sites from 2011 through 2016. The boxplots show the distribution (i.e., 25th, 50th, and 75th percentiles) of daily *LE* for each month. The whiskers indicate the 1.5 times interquartile range (25th to 75th percentile) plus the 75th percentile or minus the 25th percentile.

Evaporation lagged behind *R*_n_ by 2 months throughout the year (Figure 4). *R*_n_ peaked in June, began decreasing after the summer solstice, and reached its lowest values in December. In contrast, monthly *E* was relatively high during July–October, reaching maximal levels. Monthly *E* was relatively low during January–March and was lowest in February. Peak monthly *E* occurred in August 2015, reaching 109 mm month^−1^ at the CB site and 122 mm month^−1^ at the LI site. The lowest monthly *E* values occurred in February 2015, measuring 9 mm month^−1^ at the CB site and 7 mm month^−1^ at the LI site.

We discerned observable *E* during the ice‐free and ice‐present periods alike in winters. Ice‐free periods possessed a mean daily *E* of 2.4 and 2.3 mm day^−1^ at the CB and LI sites, respectively. Ice‐present periods had mean daily *E* values of 1.8 and 1.7 mm day^−1^ at the CB and LI sites, respectively. *E* during the ice‐free and ice‐present winter periods corresponded to ~32% and ~24% of *E* during the summer peak (e.g., 7.4 mm day^−1^ in July).

To quantify the time lag between *LE* (equivalent to *E*, with different units) and *R*_n_, we adopted a sinusoidal wave function:

(6) $$\mathrm{LE}\left(R_{n}\right)=y_{0}+A\times \sin\left(\frac{2\times \pi \times \mathrm{MOY}}{12}\right)+C,$$

where *y*_0_ represents the baseline *LE* (or *R*_n_) over the time period (MJ m^−2^ month^−1^), *A* is the seasonal amplitude that corresponds to the difference between the peak and trough of the seasonal cycle, MOY is the month of the year, and *C* is the offset term. We found

(7) $$Rn=228.16+218.77\times \sin\left(\frac{2\times \pi \times \mathrm{MOY}}{12}+2.86\right),$$

(8) $${\mathrm{LE}}_{\mathrm{CB}}=129.05+89.26\times \sin\left(\frac{2\times \pi \times \mathrm{MOY}}{12}+4.68\right),$$

(9) $${\mathrm{LE}}_{\mathrm{LI}}=121.06+101.45\times \sin\left(\frac{2\times \pi \times \mathrm{MOY}}{12}+4.68\right).$$

**Figure 5:**
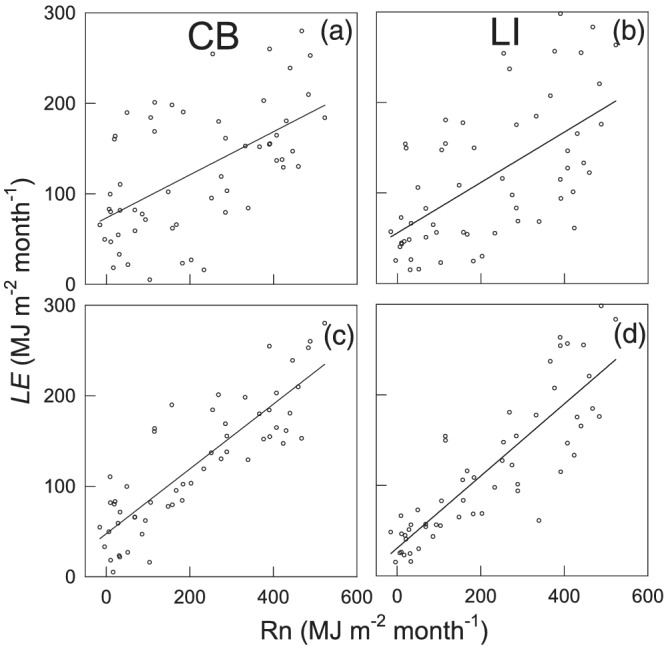
The relationships between the monthly latent heat flux (*LE*) and net radiation (*R*_n_) (a and b) at the CB and LI sites. Charts c and d show results using *R*_n_ 2 months prior to the *LE* measurements. The lines represent the linear regressions.

**Figure 6:**
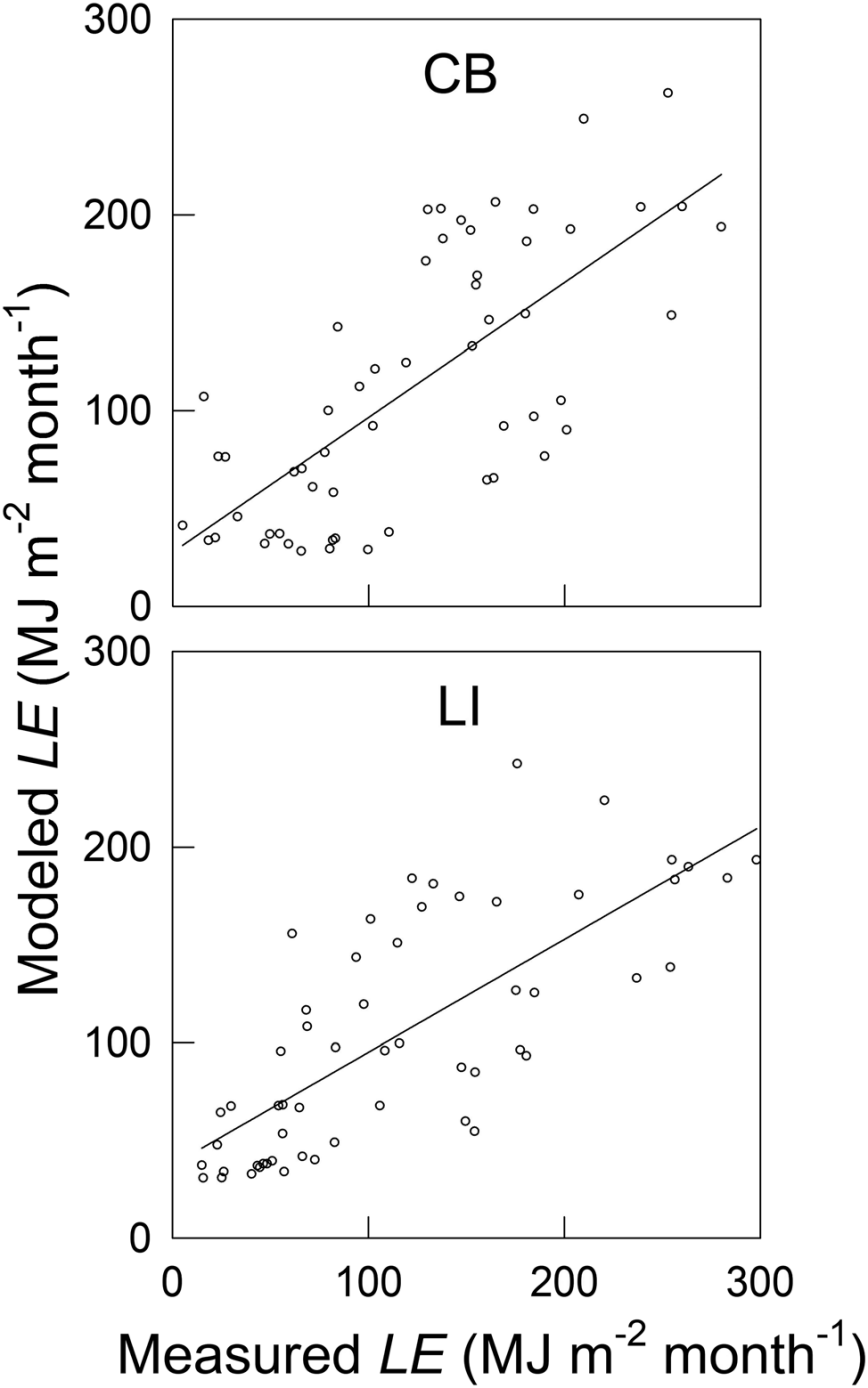
The relationship between modeled latent heat flux (*LE*, Equation 4) and measured *LE* at the CB and LI sites. All data are at a monthly scale. The lines represent the linear regressions.

The offset terms in the fitted annual sinusoidal wave models (Equations 7–9) were different between *R*_n_ and *LE*, as well as between the two sites (*p* < 0.05). The amplitude terms (*A*) were larger for *R*_n_ than for *LE* (Equation 7 vs. Equations 8 and 9) (*p* < 0.05). The baseline terms (*y*_0_) also were significantly higher for *R*_n_. In contrast, there were no significant differences between the CB and LI sites in the amplitude or the timing of peak *LE* (Equations 8 and 9). Hysteresis was evident in seasonal *LE* from *R*_n_ at both sites (Figure 4). *LE* lagged behind *R*_n_ each year. The largest correlations between *LE* and *R*_n_ were found when *LE* lagged *R*_n_ by 2 months at both the CB and LI sites (Table 3). The lag time based on the sinusoidal wave function (Equation 6) also revealed this 2‐month lag between *LE* and *R*_n_. Detailed analysis of the linear regressions between *LE* and *R*_n_ further confirmed this 2‐month lag at both sites (Figure 5; Table 3). Similar regression analyses were carried out between the measured and modeled *LE* shifted by 0–5 months. The results showed higher regression *R* ^2^ at a 1‐/2‐month shift at both sites (Figure 6; Table 4), suggesting a similar 1‐/2‐month lag between the measured and modeled *LE*. Because the Penman model did not account for heat storage in the water, the lags between *LE* and *R*_*n*_ and between measured and modeled *LE* can be attributed to the water heat storage.

**Table 3:** Analyses of the Temporal Lags Between Latent Heat Flux (*LE* (MJ m^−2^)) and Net Radiation (*R*_n_ (MJ m^−2^)) Using Shifts of 0–5 Month(s)

*E* lagged *R*_n_ months	CB				LI			
	Slope	Intercept	*R* ^2^	*p* value	Slope	Intercept	*R* ^2^	*p* value
0	0.24	73.40	0.33	<0.01	0.28	55.75	0.37	<0.01
1	0.36	47.52	0.73	<0.01	0.40	30.63	0.74	<0.01
2	0.37	46.43	0.77	<0.01	0.41	29.79	0.76	<0.01
3	0.29	63.02	0.49	<0.01	0.32	49.47	0.47	<0.01
4	0.14	96.15	0.11	0.01	0.13	89.28	0.08	0.03
5	−0.04	137.38	0.01	0.50	−0.07	135.11	0.02	0.28

*Note*. The equation is *LE* = Slope ×*R*_n_ + Intercept.

**Table 4:** Analyses of the Temporal Lags Between Measured Latent Heat Flux (*LE*_mea_ (MJ m^−2^)) and Modeled Latent Heat Flux (*LE*_mol_ (MJ m^−2^)) Using a Shift of 0–5 Month(s)

*E*_mea_ lagged *E*_mol_ months	CB				LI			
	Slope	Intercept	*R* ^2^	*p* value	Slope	Intercept	*R* ^2^	*p* value
0	0.76	39.22	0.52	<0.01	0.97	14.76	0.56	<0.01
1	0.93	19.72	0.79	<0.01	1.18	5.95	0.82	<0.01
2	0.88	26.30	0.70	<0.01	1.09	4.41	0.70	<0.01
3	0.62	55.58	0.36	<0.01	0.72	43.32	0.31	<0.01
4	0.19	107.41	0.03	0.21	0.15	104.48	0.01	0.43
5	−0.27	163.19	0.07	0.10	−0.44	167.96	0.11	0.10

*Note*. The equation is *E*_mea_ = Slope × *E*_mol_ + Intercept.

### 3.4 Physical and Environmental Regulations on *E*

**Figure 7:**
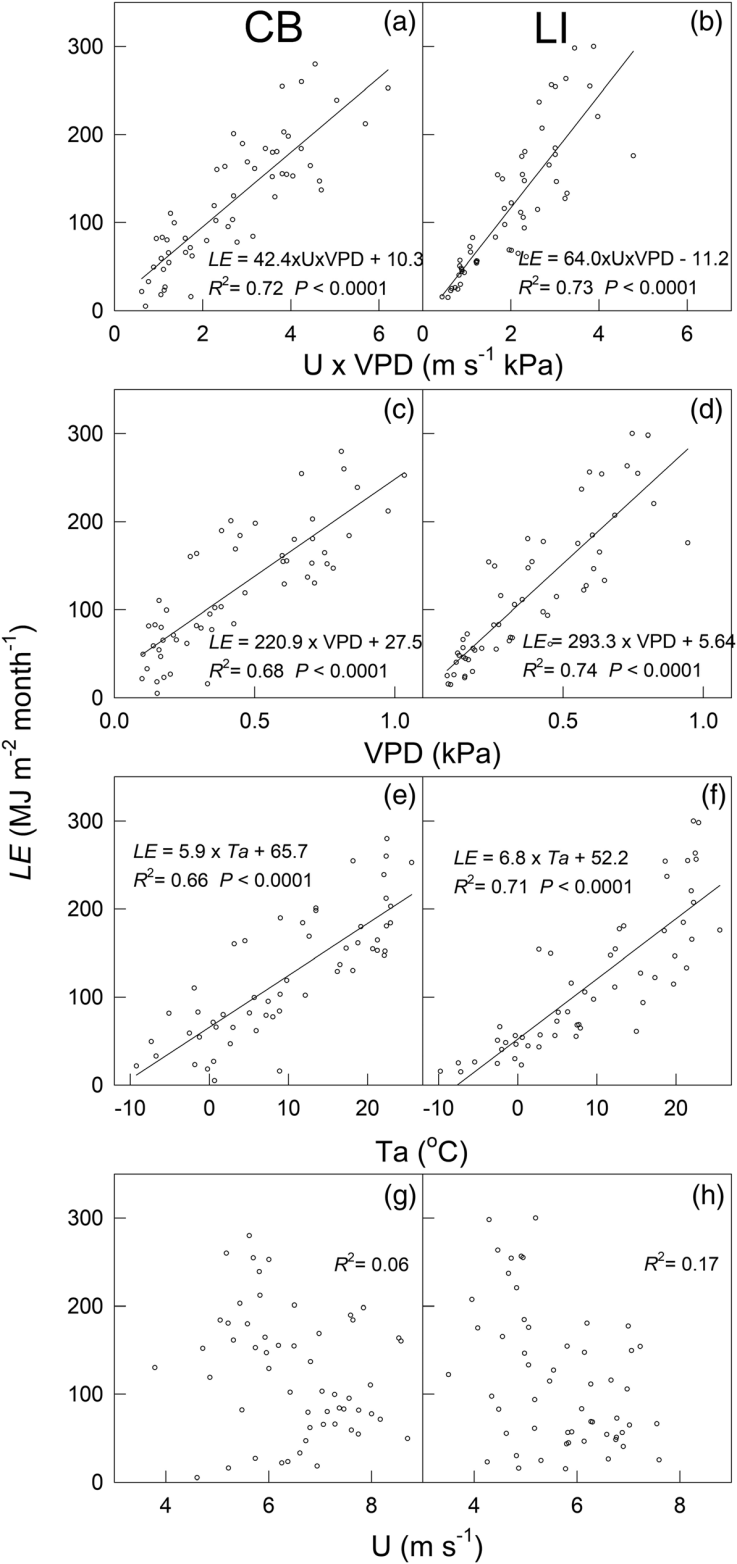
The relationships among latent heat flux (*LE*) and (a, b) the product of *U* (wind speed) and vapor pressure deficit (*VPD*), (c, d) *VPD*, (e, f) air temperature, and (g, h) wind speed at the CB and LI sites, at a monthly scale. The lines represent the linear regressions. The linear regression statistics are shown for each plot.

Significant correlations occurred between monthly *E* and several meteorological factors, including the products of *U* and *VPD* (i.e., *U* × *VPD*), *VPD*, and *T*_a_ (Figures 7a–7f), whereas *E* was uncorrelated with *U* at either site (Figures 7g and 7h). On a monthly scale, *U* × *VPD* explained ~72–73% of the variation in *E* (Figures 7a and 7b) at both sites. *T*_a_ explained ~66% and ~71% of the respective variances in *E* at the CB and LI sites (Figures 7e and 7f).

*E* during the winter period (October–March) accounted for <30% of annual *E* (Table 5), with monthly *E* values ranging from 20 to 280 mm. LE/*R*_n_ was 57% at CB and 54% at LI. Annual *E* was 635 ± 42 mm at CB and 604 ± 32 mm at LI, while during the winter period (October–March), *E* was 189 ± 61 mm at CB and 178 ± 25 mm at LI (Table 5). On an annual basis, *E* removed 620 mm of water from the lake surface annually, which is equivalent to 81% of the annual rainfall of ~770 mm.

**Table 5:** Summary of Annual (October–September) and Winter (October–March) Evaporation *E* (mm) at the CB and LI Sites

Year	CB			LI		
	Winter annual %			Winter annual %		
2011–2012	108	583	19%	139	620	22%
2012–2013	209	619	34%	180	632	28%
2013–2014	226	618	37%	178	553	32%
2014–2015	220	644	34%	173	581	30%
2015–2016	298	709	42%	218	636	34%
Average	189	635	30%	178	604	29%

### 3.5 Interannual and Between‐Site Variation in Lake *E*

Annual *E* was higher in the warmer years (e.g., first, second, and fifth) than in the cooler years (e.g., third and fourth). On the other hand, winter *E* did not show similar interannual variation between the warm and cold winters. Winter *E* was the highest in the fifth winter (the second warmest of the five winters) and the lowest in the first winter (the warmest). Noticeably, longer lags between *E* and *R*_n_ occurred in the years having cold winters (Figure 4).

Comparison of *E* between the two sites revealed evident differences, both seasonally and interannually. The nearshore site experienced higher *E* than the offshore site in winter through spring months (Figure 3, October–June), with the differences reversed in the summer (i.e., lower evaporation; Figure 3, July–September). Altogether, 5‐year *E* was 5.0% higher (given the ~3.7% uncertainty level) nearshore than offshore. Because all key meteorological factors (e.g., *U* × *VPD*, *VPD*, and *U*) were higher nearshore than offshore (Figure 2), it appeared that the between‐site *E* difference was mainly driven by climatic difference, influenced by proximity to land. Additionally, since the offshore site was located in a relatively deeper part of lake, seasonality of the between‐site difference might partly reflect difference in water heat storage.

## 4 Discussion

### 4.1 Factors Regulating Lake *E*

Monthly *E* over western Lake Erie primarily is controlled by the product of *U* and *VPD* (i.e., *U* × *VPD*), *VPD*, and *T*_a_ (Figures 7a–7f). Our findings support the assumptions of previous studies that variation in lake *E* can be largely explained by *U* × *VPD* (Blanken et al., 2000) or *VPD* (Nordbo et al., 2011). The dependency of *E* on *U* × *VPD* indicates that lake *E* is controlled jointly by the intensity of turbulent mixing and the lake‐air vapor pressure gradient (Hostetler et al., 1993; Liu et al., 2012). At Lake Erie, higher *U* occurred mostly in winter months, while higher *VPD* occurred mostly during summer months (Figure 2). Consequently, higher *U* × *VPD* occurred mostly in July–October, which explained, at least partially, higher lake *E* in the late summer and fall. The positive correlation between *E* and *T*_a_ could be explained by the dependency of lake *E* on heat transfer (Oke, 1987). Yet, since *T*_a_ and *VPD* are correlated, we cannot rule out the indirect effects of *T*_a_ via changes in saturation vapor pressure. Notably, for every 1°C increase in surface air temperature, the surface water temperature increases by 0.88°C to maintain the energy flux balance (Morrill et al., 2005).

Although *E* was not correlated with *U* on a monthly scale (Figures 7g and 7h), we found that *E* was significantly correlated with *U* at daily and hourly scales (Shao et al., 2015). In principle, higher *U* leads to increased mechanically generated turbulent mixing, as indicated by increased friction velocity, and thus increased Reynolds stress. However, increases in mechanical mixing may not always lead to increased *E*, unless other environmental conditions (e.g., available energy and moisture gradients) are also suitable for *E*. Therefore, it is not surprising that we found no evident correlation between *E* and *U* at the monthly scale.

### 4.2 Interannual and Seasonal Variation of Lake *E*

t is of interest to note that warmer years tend to have higher annual *E* for Lake Erie but such temperature dependency does not hold for winter *E*. The warmest winter (2011–2012) did not have the highest winter *E* among the five winters. Three consecutive winters (i.e., 2012–2013, 2013–2014, and 2014–2015), despite their temperature differences, possessed very similar winter *E* values at both sites. Noticeably, we did not observe similar dependency of winter *E* on ice‐present duration, as reported by previous studies (e.g., Blanken et al., 2011; Duan & Bastiaanssen, 2017; Moukomla & Blanken, 2017). The interannual variation of winter *E* cannot be explained by differences in the winter environmental conditions alone. Heat storage effect, as indicated by the 2‐month lags between *E* and *R*_n_, plays an essential role in regulating winter *E* because at least a portion of the energy sources for winter *E* is from the stored energy in the water from the summer's *R*_n_. We discerned significantly lower *E* (25%) during April and May following the coldest winters (2013–2014 and 2014–2015) than in April and May following the warmer ones (2011–2012, 2012–2013, and 2015–2016). These results also provide clues as to whether extremely warm/cold winters would lead to an increase or decrease in winter *E*.

Monthly and seasonal variations in lake *E* are affected significantly by the lake's heat storage, which primarily is determined by water depth. In temperate regions, increased *R*_n_ typically warms the water body during spring and summer (Lofgren & Zhu, 2000). During fall and early winter, as *R*_n_ decreases, the water body cools as its stored energy is released. The effect of heat storage is that water temperatures could be higher than air temperatures during fall and winter, and vice versa during summer, leading to higher *E* in fall and winter than in summer for large, deep lakes. Yet the lag time of this late‐season *E* depends on a lake's water depth and heat storage capacity, with a longer lag typical in a deeper lake (Lofgren & Zhu, 2000). On the other hand, it is suggested that the heat storage effect can be ignored for relatively shallow water bodies (e.g., <0.5 m) and reaches a maximum (i.e., the seasonal *E* ceases to change) at >4.5 m due to the fact that little incoming solar radiation penetrates beyond (Finch & Calver, 2008). We showed that Lake Erie—the shallowest and most southern of the Great Lakes—had the shortest time lag of ~2 months between the peak *R*_n_ and *E*, in contrast to ~5 months in the more northern and deeper Great Lakes. It is important to incorporate this lake‐specific thermal lag to model the seasonality of lake *E* correctly (Duan & Bastiaanssen, 2017).

### 4.3 Comparison and Upscaling of *E*

Our study provided the first direct and multiyear measurements of *E* over western Lake Erie, with annual *E* of 635 ± 42 mm and 604 ± 32 mm at the nearshore (CB) and offshore (LI) sites, respectively. Previous studies based on modeling and indirect approaches (e.g., energy budget, water balance, and mass transfer) reported a wide range of annual *E* over Lake Erie, on the order of 500–1,000 mm (Derecki, 1976; Do et al., 2020; Gronewold et al., 2019; Lofgren & Zhu, 2000; Moukomla & Blanken, 2017; Neff & Nicholas, 2005). We advocate that the *E* estimates need to be better constrained, especially those based on indirect approaches that often substantially overestimate *E* over Lake Erie (e.g., 898–903 mm) (Derecki, 1976; Neff & Nicholas, 2005; Schertzer, 1987). It is worth mentioning that even the most recent estimates using the state‐of‐the‐art model continue to overestimate annual *E* by around 200–350 mm over Lake Erie (e.g., Do et al., 2020; Gronewold et al., 2019), which again highlight the need for further assessing, validating, and refining the models using a network of eddy‐covariance towers to improve *E* predictions over the Great Lakes (Charusombat et al., 2018).

We showed that peak monthly *E* occurred mostly from August to October at Lake Erie and the winter months (October–March) accounted for near 30% of annual *E*. Such seasonality of *E* is distinct from other Great Lakes, which showed peak monthly *E* in December–January and that the majority of annual *E* was contributed during the winter months (Cleave et al., 2014; Do et al., 2020; Lofgren & Zhu, 2000; Moukomla & Blanken, 2017). The unique seasonality of *E* over Lake Erie likely is due to its bathymetry, latitude, and climate (Table 6). Particularly, water heat storage plays a critical role in regulating the seasonal dynamics of *E* (Duan & Bastiaanssen, 2017; Lofgren & Zhu, 2000). Relatively shallow Lake Erie, especially in the western basin, has less heat storage capacity (Schertzer, 1987; Vanderkelen et al., 2020). Water temperature and heat storage decrease more quickly in Lake Erie in the fall in comparison with the other Great Lakes (Mason et al., 2016). Thus, peak *E* occurred in August–October at Lake Erie, much earlier than the December–January peak in the other Great Lakes (Lofgren & Zhu, 2000; Moukomla & Blanken, 2017). Since they lacked direct *E* measurements, previous studies often have misrepresented the seasonal dynamics of *E* over Lake Erie (Derecki, 1976; Lofgren & Zhu, 2000; Moukomla & Blanken, 2017). We showed that this lake‐specific lag is critical to accurately simulate *E* and its seasonal dynamics in Lake Erie.

**Table 6:** Summary of Reported Evaporation (*E*), Latent Heat (LE), and Lake Characteristics Based on Eddy‐Covariance (EC) Measurements

Lake	Latitude	Area (km^2^)	Depth (m)	Flux measurement period	EC height (m)	Min offshore/fetch (km)	Min hourly *LE* (W m^−2^)	Max hourly *LE* (W m^−2^)	Mean hourly *LE* (W m^−2^)	Min daily *E* (mm day^−1^)	Max daily *E* (mm d^−1^)	Daily *E* (mm day^−1^)	Annual *E* (mm year^−1^)	Source
Great Slave Lake, Canada	61.92°N	27200	41	24 Jul to 10 Sep 1997 22 Jun to 26 Sep 1998	6.9	12 12				−2.0^a^ −3.9^a^	6.0^a^ 9.4^a^	1.06 1.33	386^b^ 485^b^	Blanken et al. (2000)
				12 Jun to 15 Dec 1999		12						1.82^b^	417^b^	
Ross Barnett Reservoir, USA	32.44°N	130	5	1 Sep 2007 to 31 Jan 2008	4	2			80	1.4^c^	3.9^c^		399^b^	Liu et al. (2009)
						2								Liu et al. (2012)
Valkea‐Kotinen Lake, Finland	61.14°N	0.041	2.5	Apr–Oct 2005–2008	1.5	0.035	2	120	120					Nordbo et al. (2011)
				Apr–Nov 2003		0.035								Vesala et al. (2006)
Lake Superior, USA	47.18°N	82100	5	Oct 2008 to 30 Sept 2009 1 Oct1 2009 to 30 Sept 2010 1 Jun 2008 to 2 Apr 2012	32.4	39		552			2.05	1.27 1.77 1.90	464 645 749	Blanken et al. (2011) Spence et al. (2013)
Lake Erie, USA‐CB site	41.72°N	25700	4.8	Sep 2011 to May 2016	15	4	10^c^	100^c^	48	0.28^c^	3.87^c^	1.74	634	This study
Lake Erie, USA‐LI site	41.83°N	25700	7.5	Sep 2011 to May 2016	15	12	11^c^	115^c^	50	0.20^c^	4.05^c^	1.65	604	

*Note*. Blanks = data not available.

^a^ Estimated value from their figures.

^b^ The results were from the measurement periods.

^c^ Monthly mean values.

Comparisons with the terrestrial ecosystems in the same region indicate that lake *E* is higher than those reported from local soybean (487–580 mm year^−1^) and corn croplands (539–639 mm year^−1^) (Abraha et al., 2016). Lake *E* is comparable to reported *E* from an oak‐dominated forest (578–670 mm year^−1^) (Xie et al., 2016) and is slightly lower than *E* from a coastal marsh (628–888 mm year^−1^) (Chu et al., 2015). Comparable annual evapotranspiration/evaporation between Lake Erie and nearby terrestrial ecosystems suggests that the region's evaporative water loss generally is energy limited rather than water limited. Based on spatial coverage of the region's croplands (~70%) and forests (~7%) (Chu et al., 2015), with the lake watershed estimated to be 10 times larger than the lake surface area, water loss from Lake Erie is equivalent to the total evapotranspiration from all of the forests or from 1/3 of the croplands in the watershed.

Whereas Lake Erie's annual *E* is comparable to that from the surrounding forests and croplands, the seasonal dynamics of *E* differ between the lake and terrestrial ecosystems. Both forests and croplands experience maximum evapotranspiration in June–August, while Lake Erie undergoes maximum *E* 2 months later, during August–October. The delayed peak *E* from the lake in the fall plays an essential role in the region's water cycling and thus in regulating these climate regimes.

## 5 Conclusions

Evaporation over western Lake Erie was measured at nearshore and offshore eddy‐covariance sites from 2011 through 2016. Annual evaporation was 635 ± 42 (±SD) and 604 ± 32 mm year^−1^ at the nearshore and offshore sites, equating to ~81% of the annual rainfall. Maximum monthly evaporation occurred during August–October. Winter evaporation (October–May) accounted for nearly 30% of the annual evaporation. Monthly evaporation was mainly controlled by the product of wind speed and vapor pressure deficit. Additionally, water heat storage, indicated by a 2‐month lag between peak net radiation and evaporation, also modulated the seasonal dynamics of evaporation. This 2‐month lag was much shorter than the ~5‐month lag reported for the other (deeper) Great Lakes. The difference in *E* between our offshore and nearshore sites suggested that within‐lake spatial heterogeneity likely contributed to this climatic and bathymetric difference. Our findings highlight the need for climate and lake models to incorporate this thermal time lag and within‐lake heterogeneity to accurately simulate the seasonal dynamics of region‐scale evaporation.

## Data Availability

All data for this paper are available from the free open access repository (at http://doi.org/10.5281/zenodo.3859063), accessed 2020‐7‐27, reproduced from Zenodo.
